# Disproportionate impact of the COVID-19 pandemic on socially vulnerable communities: the case of Jane and Finch in Toronto, Ontario

**DOI:** 10.3389/fpubh.2025.1448812

**Published:** 2025-06-11

**Authors:** Zahra Movahedi Nia, Cheryl Prescod, Michelle Westin, Patricia Perkins, Mary Goitom, Kesha Fevrier, Sylvia Bawa, Jude Dzevela Kong

**Affiliations:** ^1^Resilience Research Atlantic Alliance on Sustainability, Supporting Recovery and Renewal (REASURE2) Network, Toronto, ON, Canada; ^2^Africa-Canada Artificial Intelligence and Data Innovation Consortium (ACADIC), Department of Mathematics and Statistics, York University, Toronto, ON, Canada; ^3^Global South Artificial Intelligence for Pandemic and Epidemic Preparedness and Response Network (AI4PEP), Toronto, ON, Canada; ^4^Black Creek Community Health Centre, (BCCHC), Toronto, ON, Canada; ^5^Faculty of Environment and Urban Change, York University, Toronto, ON, Canada; ^6^School of Social Work, York University, Toronto, ON, Canada; ^7^Department of Geography and Planning, Queen's University, Kingston, ON, Canada; ^8^Department of Sociology, York University, Toronto, ON, Canada; ^9^Artificial Intelligence and Mathematical Modeling Lab (AIMM Lab), Dalla Lana School of Public Health, University of Toronto, Toronto, ON, Canada; ^10^Institute of Health Policy, Management and Evaluation (IHPME), University of Toronto, Toronto, ON, Canada; ^11^Bahen Centre for Information Technology, Department of Mathematics, University of Toronto, Toronto, ON, United States

**Keywords:** COVID-19, greater Toronto area, Gini index, Jane and Finch community, local indicator of spatial association, statistical tests, socially vulnerable population

## Abstract

**Objective:**

This work aims to study the disproportionate impact of the COVID-19 pandemic on the Jane and Finch community, one of the socially vulnerable neighborhoods in the Greater Toronto Area (GTA), Ontario, Canada, in terms of morbidity, mortality, and healthcare services.

**Methodology:**

A dataset provided by the Black Creek Community Health Centre (BCCHC), gathered from different health-related portals, covering various health statistics during COVID-19, namely, COVID-19 number of cases, hospitalizations, deaths, percentage of vaccination with one-, two-, and three-dose(s), Primary and Preventive Care (PPC) visits which include fecal and pap-smear cancer tests, and percentage of completed Imaging, Procedures, and Surgeries (IPS) which include the number of patients waiting for surgery were studied using statistical analysis. Underserved communities in the Peel, York, and City of Toronto regions were recognized using the Ontario Marginalized Index (ON-Marg). The Jane and Finch community was selected from the fifth quintile of the ON-Marg index and compared with the remaining locations (first to fourth ON-Marg quantiles) using Kruskal-Wallis, Mann–Whitney u, and *t*-tests. The Gini index was used to understand the inequality of the health parameters among the selected neighborhoods. Local Indicator of Spatial Association (LISA) was used to detect the neighborhoods with significantly higher numbers of COVID-19 cases, hospitalizations, and mortalities.

**Results:**

The Jane and Finch community had a significantly (*p* < 0.0001) higher number of COVID-19 cases, hospitalizations, and mortalities. The significance and cluster analysis of LISA also extracted the Jane and Finch community as one of the hotspots with significantly higher COVID-19 infection, hospitalization, and death. The percentage of the third-dose vaccination was significantly lower for the Jane and Finch community (*p* = 0.0004). The number of patients during the COVID-19 pandemic versus before that decreased significantly more for pop-smear tests (*p* = 0.041) and surgery waitlists (*p* = 0.037) for the Jane and Finch community.

**Conclusion:**

As one of the most socially vulnerable communities of GTA, the Jane and Finch community has endured a heavier burden of the disease during the COVID-19 pandemic. This work aims to help the Jane and Finch community recover faster by shedding light on health areas in which it has suffered more from the COVID-19 outcomes.

## 1 Introduction

The first cases of the novel coronavirus 2019 (COVID-19) were observed in Wuhan, China in December 2019 and soon after in other nations around the globe. Countries successively declared a state of emergency and applied Non-Pharmaceutical Interventions (NPI) to contain the virus. Although NPIs successfully decreased the spread of the virus, they significantly reduced the number of non-urgent clinical demands, healthcare scanning, tests, operations, and surgeries, causing a dramatic increase in the demand/waitlist (1, 2). The COVID-19 pandemic and lockdowns have disproportionately affected underserved communities, which include groups facing social and structural disadvantage. These populations are often described using overlapping but distinct terms: “disadvantaged” refers to those who face structural barriers to opportunities or well-being, “deprived” populations lack essential resources such as stable income, housing, education, or healthcare, “vulnerable” groups are at increased risk of adverse health outcomes due to pre-existing conditions or social determinants of health, and “marginalized” populations are excluded from full participation in economic, political, cultural, or social life (3). These underserved groups have historically borne a heavier burden of illness, mortality, and healthcare disruption during public health crises (4). The same pattern has been observed again during the COVID-19 pandemic (5, 6). Studying the health inequality of underserved populations is important for determining and applying preventive measures in future crises and pandemics. It is therefore essential to comprehensively understand and document the disproportionate impact of the COVID-19 pandemic on these populations. While some prior studies have examined this issue, the scope of research on the pandemic’s effects on these communities remains limited (7–9). Harlem (10) conducted a descriptive analysis to compare six community districts of Queens, New York. Three of the communities had an extremely high number of COVID-19 cases, and the other three had a moderate number of cases. The results show that equity metrics such as overcrowding, lower education, less access to healthcare, and more chronic diseases significantly affected the number of cases in different communities. McCann, et al. (11) found that substance use increased among street-involved individuals during the COVID-19 pandemic. Siedner, et al. (12) have studied the impact of lockdowns on the primary healthcare system of rural South Africa. The results show that adult ambulatory care visits were maintained throughout the pandemic. However, child healthcare visits had reduced during the early pandemic but increased to the pre-COVID-19 number afterwards. Their modeling analysis suggests that this will significantly increase mortality in children. Moreover, according to the World Health Organization (WHO), malaria cases among children under 5 years old have risen substantially in endemic regions where access to malaria care and insecticide-treated nets was disrupted.

Nayak, et al. (13) employed generalized linear mixed models, and found that the Case Fatality Rate (CFR) of COVID-19 in states of the USA with high Social Vulnerability Index (SVI) is 63% higher than those with low SVI. Authors in (14) have studied access to health sites such as respiratory, gastric, waterborne and mosquito-borne illnesses, and hypertension in slums of Bangladesh, Kenya, Nigeria, and Pakistan and found a decline in access to healthcare across all sites during the early stages of the COVID-19 pandemic. Additionally, they reported an increase in healthcare costs alongside a reduction in household income. In (15) the effect of 46 variables across five different categories, namely, access to medical services, underlying health conditions, environmental exposure, vulnerability to natural disasters, and sociodemographic, behavioral, and lifestyle factors were studied on disparate populations of Harris County, Texas. After applying Principal Component Analysis (PCA) for dimension reduction, the results of the rank-based exceedance method and k-means clustering indicated that all five categories were effective in increasing the number of COVID-19 cases among socially vulnerable populations. In (16) using a quantitative rapid review, the availability of health communication in most the common native languages of migrant groups across 47 European countries was studied. The results show that 48% of countries had information in at least one migrant language. However, in only 6% of the countries, healthcare entitlements were translated into common migrant languages. This showed that in most countries, COVID-19 preventive measures were not considered or properly explained to the immigrants, one of the most vulnerable groups in European countries. The present study focuses on the impact of the COVID-19 pandemic on the Jane and Finch community, one of the marginalized and disadvantaged communities of the Greater Toronto Area (GTA), which faces systematic barriers to resources, resulting in increased vulnerability to adverse health outcomes and material deprivation. To date, this specific context has not been adequately studied.

Jane and Finch is located in the North York district, at the northwest end of Toronto, Ontario, near the borders of York Region and Peel Region. It is a highly multicultural and diverse neighborhood with approximately 61% of residents being immigrants or refugees, and 77–81% identifying as visible minorities (17, 18). The area is also characterized by low income and high population density (19). Peel Region, known for its rapid urban growth, is ethnically diverse and has a large immigrant and visible minority population. York Region features a mix of urban and suburban areas, increasing ethnic diversity, and pockets of income inequality. Toronto, Canada’s largest city, displays wide variations in wealth and health outcomes across neighborhoods. Peel and York Regions, along with other areas of Toronto, were selected as comparators due to their geographic proximity to Jane and Finch, similar demographic diversity, and the presence of neighborhoods with varying degrees of social vulnerability. Including these regions provides a contextualized understanding of how structural inequities across closely situated communities influenced health outcomes during the COVID-19 pandemic.

The Jane and Finch community was primarily developed in the 1960s as part of Toronto’s broader post-war suburban expansion. Originally a rural area, Jane and Finch underwent rapid urbanization following the construction of major infrastructure such as Highway 400 in 1952 and the establishment of Metropolitan Toronto in 1953. The neighborhood was designed with a mix of private and public housing, with a significant proportion of publicly funded housing projects built by the Ontario Housing Corporation to accommodate Toronto’s growing and increasingly diverse population. By 1971, the area’s population had grown from roughly 1,300 a decade earlier to over 33,000, with over 20% of its housing stock designated as public housing—far above the North York average. This early development shaped the socioeconomic structure of the community, contributing to its status today as one of Toronto’s most diverse yet underserved urban neighborhoods (20).

**Figure 1:**
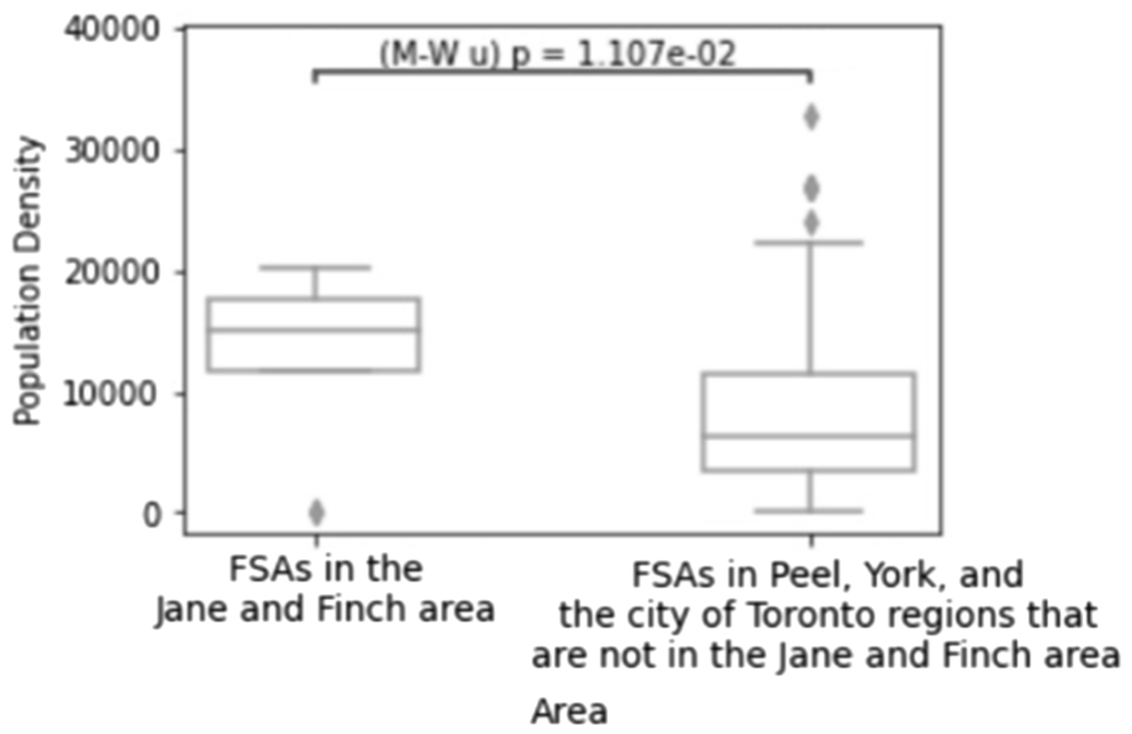
Population density of the FSAs in the Jane and Finch community compared to other FSAs located in Peel, York, and City of Toronto. Created using Python.

Figure 1 compares the distribution of the population density of all Forward Sortation Areas (FSAs) in the Jane and Finch community with other FSAs in Peel, York, and the city of Toronto. The Mann–Whitney U test *p*-value (=0.01107) indicates that Jane and Finch has a significantly higher density compared to other neighborhoods in Peel, York, and the City of Toronto.

High population density reportedly accelerates the spread of the coronavirus and results in more hospitalizations and fatalities (21–24). In (25), using Pearson correlation coefficients, it has been discovered that population density has a great impact on COVID-19 cases in four of the five states under study in India. The correlation is validated using the response surface methodology and Thiessen polygon approaches. Authors in (26) used a rank-size rule test to find the impact of population density on the spread of coronavirus in various cities of Israel. The results show that when density increases, the number of COVID-19 cases rise. However, the infection rate is lower in really dense populations due to improved health infrastructure, hygienic practices, social distancing, and wearing masks. Similarly, other studies have discovered that in low-income neighborhoods of Toronto, higher population density is associated with a faster increase in COVID-19 infections (27, 28).

A limited number of studies have examined the neighborhood conditions of Jane and Finch (29). Zaami (30) conducted a survey on the socio-spatial circumstances of Ghanaian immigrants settled in Jane and Finch. She found that the predominant challenge that affects all other aspects of resident’s lives including education, employment, and finance is the neighborhood’s negative reputation linked to perceptions of high crime, youth deviance, and substance use/addiction which perpetuates social stigma. Skeete and Dippo (31) used surveys and interviews to explore challenges faced by youth in Jane and Finch. The findings revealed that the most important challenges that youth from the Jane and Finch community deal with include financial hardship, unemployment, limited access to education and training, health concernsexposure to violence, mental health issues, inadequate support for substance use and sexual health services.

In this work, the impact of the COVID-19 pandemic on settlers of the Jane and Finch community is studied through comparing the neighborhood with the other locations in Peel and York regions and the city of Toronto in the GTA. In the following, section 2 explains the methodology, section 3, describes the results found using statistical and LISA analysis, section 4 presents the discussion of our work, and section 5 includes the conclusion.

## 2 Materials and methods

### 2.1 Dataset

The dataset used in this study was compiled by the Black Creek Community Health Centre (BCCHC), a community-based primary care organization that serves residents of the Jane and Finch neighborhood in northwest Toronto. The BCCHC gathered this data from a range of provincial administrative databases, including the Ontario Health Insurance Plan (OHIP), Client and Health Related Information System, Transfer Payment Ontario, Ontario Drug Benefit Claims, Drug and Alcohol Treatment Information System, Narcotics Monitoring System, Bed Census Summary, Ontario Healthcare Financial and Statistics, and the Wait Time Information System. These are government-held data repositories that track a broad range of healthcare utilization and delivery metrics across Ontario. The dataset includes aggregated health statistics for patients residing in three Ontario public health units: Toronto (Master No. 3895), York Region (Master No. 2270), and Peel Region (Master No. 2253). The Master Nos are internal identifiers assigned by the Ministry of Health to each health unit for administrative tracking and reporting purposes (32). The ratio presented in Equation 1 was used to compare the health parameters during the COVID-19 pandemic with those before that.


(1) $$r=\frac{\text{Patients}\_\text{During}\_\text{COVID}}{\text{Patients}\_\text{Before}\_\text{COVID}}$$

Where Patients_During_COVID and Patients_Before_COVID are the number of patients during and before the COVID-19 pandemic, respectively. Therefore, when the result of Equation 1 is above/below 1, the number of patients during COVID-19 has increased/decreased compared to before that. The dataset is available upon a reasonable request from the corresponding author and with permission from the BCCHC. All patients were residents of Peel, York, and City of Toronto regions. The dataset includes three set of parameters as described below:

1. COVID-19 statistics:

- COVID-19 cases, hospitalizations, and mortalities per 1,000 people (in the first quarter of 2022).
- COVID-19 first, second, and third dose vaccination in percentage

2. Primary and Preventive Care (PPC):

- Number of primary care visits during the COVID-19 pandemic (December 2021) compared to before that (December 2019), calculated using Equation 1.
- Number of cancer fecal screenings during the COVID-19 pandemic (first quarter of 2022) compared to before that (first quarter of 2020), calculated using Equation 1.
- Number of pap-smear screenings during the COVID-19 pandemic (first quarter of 2022) compared to before that (first quarter of 2020), calculated using Equation 1.

3. Imaging, Procedures, and Surgeries (IPS):

- Number of completed surgeries during the COVID-19 pandemic (first quarter of 2022) compared to before that (first quarter of 2020), calculated using Equation 1.
- Number of completed ophthalmic treatments during the COVID-19 pandemic (first quarter of 2022) compared to before that (first quarter of 2020), calculated using Equation 1.
- Number of completed orthopedic treatments during the COVID-19 pandemic (first quarter of 2022) compared to before that (first quarter of 2020), calculated using Equation 1.
- Number of completed MRI screenings during the COVID-19 pandemic (first quarter of 2022) compared to before that (first quarter of 2020), calculated using Equation 1.
- Number of patients waiting for surgery during the COVID-19 pandemic (first quarter of 2022) compared to before that (first quarter of 2020), calculated using Equation 1.

In this study, we have treated both vaccination rates and COVID-19 outcomes (cases, hospitalizations, and deaths) as health indicators of interest in vulnerable populations. While vaccination is typically viewed as an intervention or exposure that influences COVID-19 outcomes, we analyze it here as an outcome of structural inequities, i.e., the result of access, outreach, and trust disparities across communities. Lower vaccination coverage in areas such as Jane and Finch is not merely a confounding variable but reflects the uneven distribution of public health resources and engagement efforts. Hence, in the context of this work, the vaccination rate is considered both a modifiable factor and a social outcome reflective of systemic disadvantage (33).

### 2.2 Community selection and statistical analysis

Ontario Marginalization Index (ON-Marg) which is a composite indicator for identifying underserved localities, ranks different neighborhoods based on four census measures: residential and dwelling instability, material (employment and education) deprivation, age and labour force participation, and ethnic and newcomer concentration (34, 66). Recent discussions in public health literature emphasize framing these dimensions not merely as community characteristics and marginalization, but as outcomes of broader systemic inequities such as structural racism, historical exclusion, and economic disenfranchisement which encompasses all aspects of vulnerability, deprivation, disadvantage, and marginalization. This perspective shifts the focus away from individual or community-level deficits and toward the structural conditions that produce and sustain health inequities (3, 34). The ON-Marg index is commonly used in quintiles, with the first and fifth quintiles representing the least and most socially vulnerable populations, respectively.

**Figure 2:**
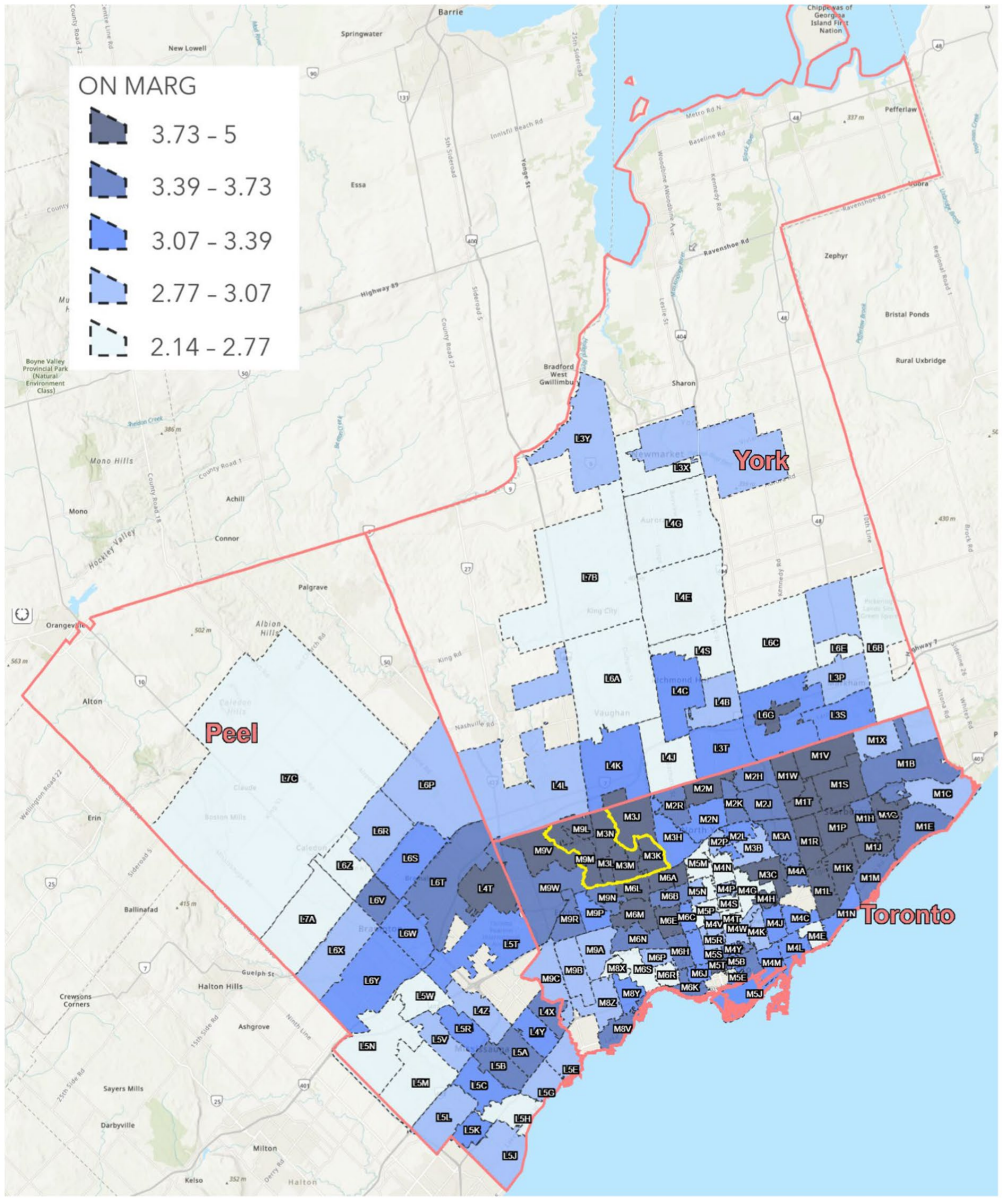
Average ON-Marg index for all the FSAs under study in the Peel, York, and City of Toronto regions. Created using ArcGIS Pro.

In this work, to study the negative impact of the COVID-19 pandemic on underserved populations, we focus on the Jane and Finch community as a case study and compare it to neighborhoods with lower levels of social vulnerability. Figure 2, created using ArcGIS online (35), displays the FSAs under study within the city of Toronto, Peel, and York regions of GTA, categorized by their ON-Marg quintile. In Figure 2, the dashed and the pink borders define different FSAs and regions, respectively. The Jane and Finch area composing seven FSAs, namely, M3J, M3M, M9L, M3N, M9M, M3L, and M3K, is highlighted with a yellow border (36).

Unfortunately, it is clear from Figure 2 that the FSAs of the Jane and Finch area are placed in the fifth quintile of the average ON-Marg index. We compare the inequality in the health parameters of the FSAs in the Jane and Finch neighborhood with FSAs that are classified in the first to fourth quintiles of the average ON-Marg composite index, namely available in Supplementary file 1.

The Gini index, which is an indicator between 0 and 1, calculates inequality based on the Lorenz curve (37, 38). Typically, when the Gini index is lower than 0.2, between 0.2 and 0.3, between 0.3 and 0.4, and between 0.4 and 0.5, and higher than 0.5, the data has perfect equality, relative equality, adequate equality, big inequality, and severe inequality, respectively (39). We first use the Gini index to understand the inequality of COVID-19 burden over all the FSAs from all the ON-Marg classes. Next, we compare different health parameters of Jane and Finch community with less socially vulnerable communities (first to fourth quantile of ON-Marg index) on FSA-level using *t*-test, Kruskal Wallis test, and Mann Whitney U test. The difference between the *t*-test and Kruskal Wallis test is that the *t*-test evaluates the difference between two distributions using their mean value, while the Kruskal Wallis test does that using their median. Therefore, the Kruskal Wallis test could be advantageous to the *t*-test, since it is less affected by outliers. In addition, Mann Whitney U test is used to verify if values from the Jane and Finch community are significantly higher or lower than the rest of the neighborhood. Most often, in statistical tests, a *p*-value lower than 0.05 is considered significant (40).

Finally, the Local Indicator of Spatial Association (LISA) is used to understand which FSAs have suffered the most in terms of COVID-19 cases, hospitalizations, and mortalities. LISA which is commonly used for detecting hotspots, includes statistics for identifying variations over geographical regions when the spatial relationship of the variable is not constant across the area under study (41). LISA mainly includes two indicators, namely, significance and cluster measures. Significant analysis is used to understand which regions are significantly different from other locations in the area under study. Moreover, cluster analysis is used to discover whether the variable (i.e., COVID-19 cases, hospitalizations, mortalities) for the specific region is higher or lower compared to other neighborhoods in the area.

LISA was carried out using GeoDa, a software developed for spatial analysis (42). After uploading the shapefile in GeoDa, the weights between FSAs were determined based on the Euclidean distance between the centroids of the two FSAs. The reason for this choice was that the Euclidean distance-based weights provided the smallest Akaike Information Criterion (AIC), when the variables (i.e., COVID-19 cases, hospitalizations, mortalities) were modeled based on the ON-Marg index (42).

## 3 Results

### 3.1 Statistical analysis

Previous studies have shown that underserved communities suffer the most in terms of morbidity and mortality during outbreaks (43–45). The *p*-values in Table 1 show a significant distinction between the Jane and Finch community and other locations in the number of COVID-19 cases, hospitalizations, mortalities, and third-dose vaccination. The *t*-test statistics show that the number of COVID-19 cases, hospitalizations, and deaths is higher and the percentage of third-dose vaccination is lower in the Jane and Finch community compared to less socially vulnerable neighborhoods. Moreover, it is evident from Table 1 that the number of pap-smear screenings and surgery patients during the COVID-19 pandemic versus before that have decreased significantly more in the Jane and Finch community compared to other FSAs.

**Table 1:** Gini index of all FSAs under study and the statistical results obtained through comparing the FSAs of Jane and Finch with other FSAs under study within Peel, York, and City of Toronto regions.

Health Parameter	Gini index	*T*-test statistics	*T*-test *p*-value	Wallis-Kruskal *p*-value	Mann–Whitney U *p*-value	Mean in Jane and Finch	96% CI in Jane and Finch
CML COVID-19 cases per 1,000	0.2	6.7117	**>0.00001**	**0.000025**	**>0.00001**	15.1685	[14.4824, 15.8545]
CML COVID-19 hospitalizations per 1,000	0.29	9.8086	**>0.00001**	**0.00002**	**>0.00001**	8.4898	[6.6121, 10.3675]
CML COVID-19 deaths per 1,000	0.41	3.4822	**0.000833**	**0.000626**	**0.000178**	2.1859	[1.3722, 2.9996]
1st dose Vax %	0.028	−0.09434	0.92506	0.63968	0.64527	84.6017	[79.01168, 90.19165]
2nd dose Vax %	0.028	−1.00966	0.31552	0.1847	0.18729	80.3257	[75.79056, 84.86086]
3rd dose Vax %	0.098	−3.86123	**0.000219**	**0.000446**	**0.000459**	38.1814	[34.80514, 41.55771]
Primary care	0.036	−1.38088	0.170936	0.132103	0.134118	0.948585	[0.936848, 0.960323]
Cancer fecal	0.078	−0.47751	0.63424	0.831165	0.840786	1.30561	[1.184237, 1.426976]
Pap-smear	0.085	−1.87158	0.0647	**0.04329**	**0.04411**	1.17321	[0.936323, 1.410101]
Completed surgeries	0.118	−1.14911	0.253772	0.226977	0.230029	0.795389	[0.5977356, 0.9930439]
Completed ophthalmic	0.1781	−0.74517	0.458531	0.2981968	0.3026931	0.863811	[0.305034, 1.422588]
Completed orthopedic	0.1657	−1.34275	0.183949	0.1132925	0.1167349	0.736383	[0.585523, 0.8872432]
Completed MRI	0.0933	−0.17344	0.862716	0.9813255	0.9875497	1.00627	[0.8224024, 1.190147]
Surgery waitlist	0.0734	−2.19637	**0.030498**	**0.0307008**	**0.0312434**	0.978036	[0.8964654, 1.059606]

Significant *p*-values are bolded in this table (*p* < .05).

The *t*-test statistics of Table 1 indicate that the percentage of first- and second-dose vaccination in the Jane and Finch community is lower than in other neighborhoods, but not significantly. The reason is that as indicated by the Gini index, there is perfect equality among all the FSAs for the first- and second-dose vaccinations. Similarly, according to the *t*-test statistics of Table 1, the number of fecal cancer screenings, completed surgeries, completed ophthalmic procedures, completed orthopedic procedures, and completed MRIs during COVID-19 pandemic versus before that have (non-significantly) decreased more in the Jane and Finch community compared to other locations. Nonetheless, the Gini index shows that these health parameters have perfect equality among all the neighborhoods, from all the ON-Marg quantiles.

### 3.2 LISA analysis

**Figure 3:**
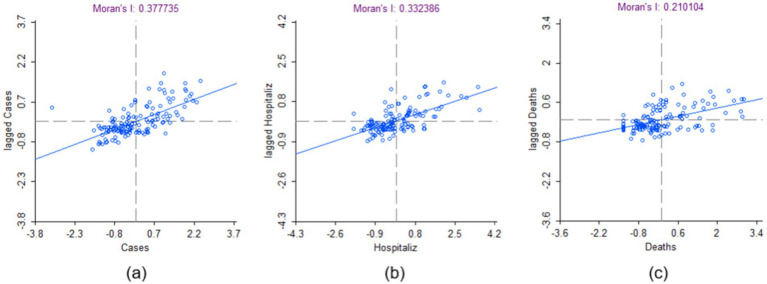
Moran’s I correlation for the cumulative number of COVID-19 **(a)** cases, **(b)** hospitalizations, and **(c)** mortalities of the FSAs under study within Peel, York, and City of Toronto regions. Created using GeoDa.

**Figure 4:**
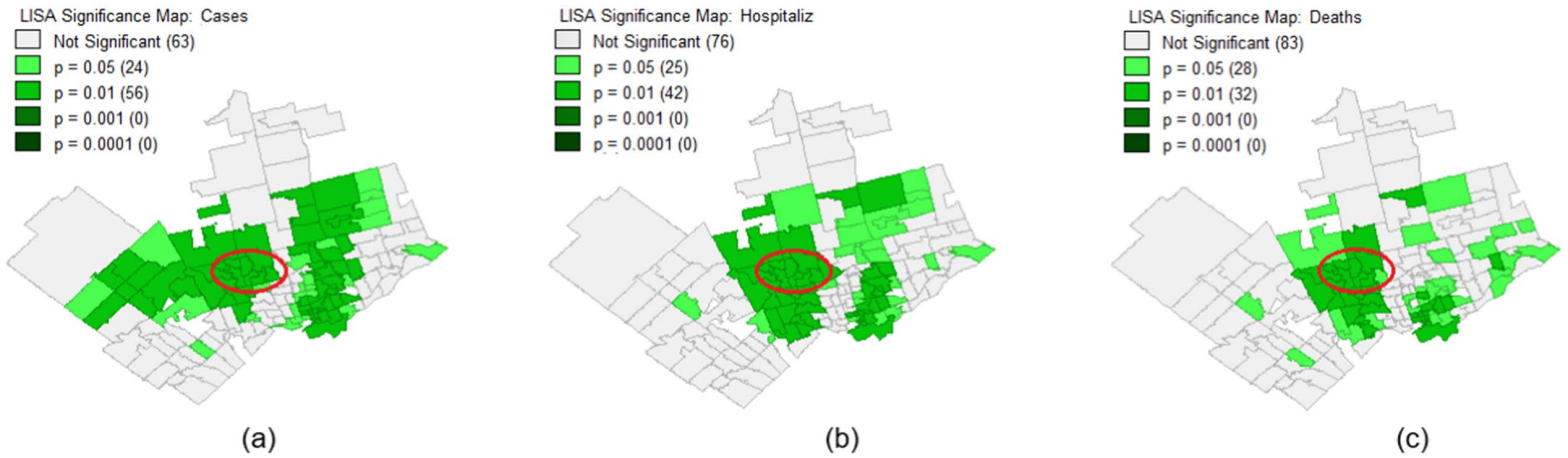
Significance map for the FSAs under study within Peel, York, and City of Toronto regions for the cumulative number of COVID-19 **(a)** cases, **(b)** hospitalizations, and **(c)** mortalities.

**Figure 5:**
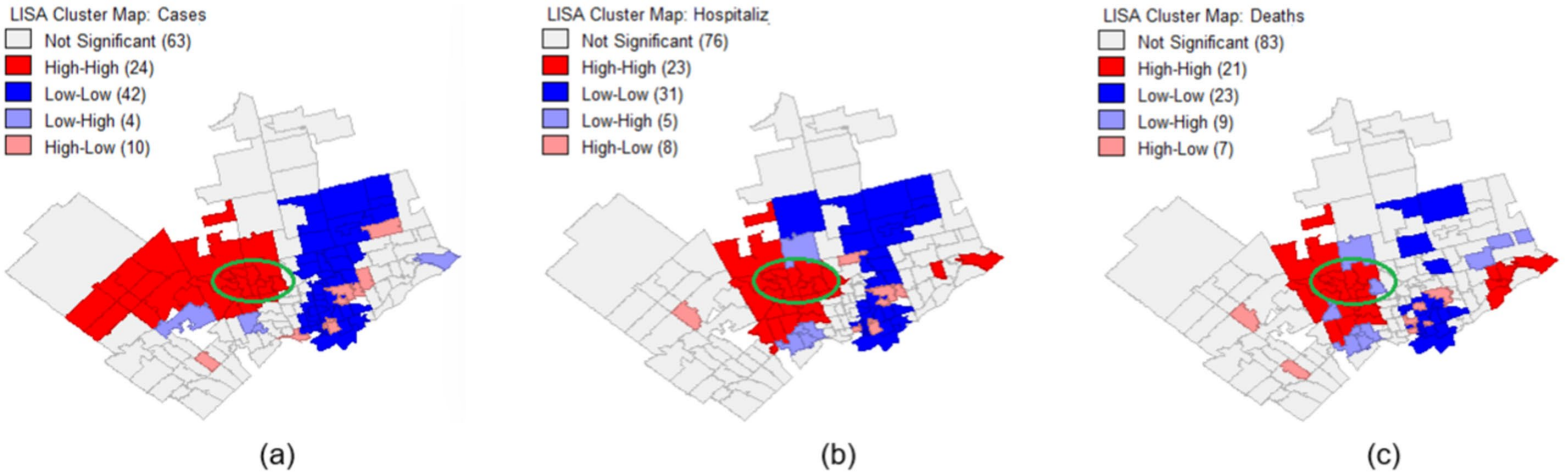
Cluster map for the FSAs under study within Peel, York, and City of Toronto regions for the cumulative number of COVID-19 **(a)** cases, **(b)** hospitalizations, and **(c)** mortalities.

Figures 3a–c show that the local Moran’s I correlation for COVID-19 cases, hospitalizations, and mortalities is equal to 0.38, 0.33, and 0.21, respectively, showcasing a significant correlation between different neighborhoods. Yet, according to the significance map displayed in Figures 4a–c for COVID-19 number of cases, hospitalizations, and mortalities, respectively, some FSAs, including the Jane and Finch community are significantly different (*p* = 0.01) compared to other neighborhoods. Moreover, Figures 5a,b show that all the FSAs in the Jane and Finch community have a significantly high number of COVID-19 cases and hospitalizations, and Figures 5c, shows that except for M3K, all other FSAs of Jane and Finch community have a significantly high number of mortalities amongst the area under study. The Jane and Finch community has been featured in the significance maps (Figures 4a–c and cluster maps (Figures 5a–c) using an oval.

Statistical analyses (Table 1) and LISA analysis (Figures 4 and 5) reveal that the Jane and Finch community experienced significantly higher rates of COVID-19 infection, hospitalization, and mortality, alongside lower vaccination uptake, particularly for the third dose. The data also show reduced access to PPC (notably pap-smear screening) and IPS (particularly surgeries), indicating disproportionate barriers to healthcare during the pandemic. More information on statistical analysis and LISA analysis of the area under study can be found in Supplementary file 1.

## 4 Discussion

This work uses statistical and spatial analysis to compare the Jane and Finch community with other neighborhoods of the Peel, York, and City of Toronto regions. The results show that the Jane and Finch community has endured a heavier burden of disease during the COVID-19 pandemic. Previous studies have also stated that underserved communities have suffered more from the COVID-19 pandemic. Nayak, et al. (13) found that a unit increase in county-level social vulnerability in the USA results in 63% increase in Case Fatality Rate (CFR). Ribeiro, et al. (46) conducted a study in Brazil and found that socioeconomic indicators such as lower educational attainment, greater household crowding, lower income, and higher population density were associated with increased COVID-19 fatality.

Karácsonyi, et al. (47) developed a risk prediction model for various cities in Australia and extracted two main factors associated with higher risk, namely, demographic vulnerability, and economic vulnerability. Daras, et al. (48) studied the impact of the COVID-19 pandemic on socially vulnerable communities in England and found that four factors, living in care homes, admission to a hospital in the past 5 years for a long-term health condition, living in overcrowded housing, and being from an ethnic minority population were directly associated with higher (age-adjusted) COVID-19 mortality rate. Our results in this study confirm the findings of previous works and the elaborate that underserved populations such as the Jane and Finch community have suffered more from the COVID-19 pandemic consequences. Studying the impact of COVID-19 pandemic on socially vulnerable communities will help inform measures and policies to protect them and improve their condition. This work focuses on one of the most socially vulnerable communities of GTA, the Jane and Finch community, to uncover the health areas that need recovery and repair. The lower COVID-19 vaccination rates observed in the Jane and Finch community contributed to significantly higher hospitalization and mortality rates. In the long term, this disparity may lead to several adverse outcomes:

- Persistent health inequalities: a study conducted by Nalbandian, et al. (49) shows that COVID-19 survivors, especially those with severe illness, are at heightened risk for developing post-acute sequelae of COVID-19 (long COVID), including chronic fatigue, respiratory dysfunction, cardiovascular disease, neurological impairments, and mental health challenges.
- Reduced workforce participation: Increased rates of long-term disability can result in lower employment rates and greater economic hardship, further destabilizing already underserved communities (50).
- Widening health disparities: Chowkwanyun and Reed (51) argue that as COVID-19-related chronic illnesses accumulate, the health gap between underserved communities (like Jane and Finch) and more affluent neighborhoods may continue to grow, exacerbating racial and socioeconomic health inequities.
- Erosion of trust in healthcare systems: previous studies (52, 53) suggest that perceived shortcomings in healthcare responses, such as ineffective vaccine outreach, can undermine trust in health institutions and negatively impact future public health initiatives.

Moreover, fewer number of patients from the Jane and Finch community have visited health clinics especially in certain health areas such as pap-smear tests and surgery, which in the long-run will have serious outcomes including:

- Disease progression and complication: delays in surgeries and health services can allow diseases to progress to more advanced, harder-to-treat stages, leading to worse prognoses and more invasive treatments (54).
- Increased morbidity and mortality: certain elective surgeries, while not immediately life-saving, significantly affect quality of life and delays can lead to increased mortality risk or permanent disability (54).
- Chronic pain and deteriorating mental health: prolonged waiting can cause or exacerbate chronic pain conditions, leading to increased depression, anxiety, and social isolation (55).
- Higher future healthcare costs: more complex and late-stage treatments are costlier, placing further financial strain on individuals and the healthcare system, especially in already underserved communities (56).

Several structural factors contribute to the disadvantage faced by residents of the Jane and Finch community. These include:

- Socioeconomic disadvantage: Higher rates of poverty, unemployment, and precarious work (e.g., essential jobs without paid sick leave) limited residents’ ability to socially distance and increased exposure to COVID-19 (57, 58).
- Overcrowded housing: Higher residential density and multigenerational living arrangements made home isolation difficult, facilitating viral transmission (59, 60).
- Healthcare access barriers: Financial constraints, language barriers, limited transportation, and distrust in the healthcare system contributed to lower healthcare utilization and vaccination rates (61).
- Systemic racism and discrimination: Structural inequities, including historical underinvestment in public services in marginalized neighborhoods, exacerbated existing health disparities (62, 63).

Our results indeed show that residents of the Jane and Finch area, accessed significantly fewer PPC and IPS services such as pap-smear and surgeries during the COVID-19 pandemic. This reduction can be attributed to several interrelated factors documented in the literature (64):

- Healthcare system strain and delays: hospitals and clinics prioritized COVID-related care, leading to postponed or canceled non-urgent appointments and elective surgeries.
- Digital divide and telehealth inaccessibility: many underserved populations lacked access to reliable internet, digital literacy, or devices necessary for virtual appointments.
- Fear and mistrust: Fear of contracting COVID-19 in healthcare settings, combined with historical mistrust of the medical system, may have discouraged people from seeking care.

Other factors may also explain the heightened COVID-19 burden observed in the Jane and Finch community. Previous research has shown that residents in this area are more likely to be employed in essential, front-line occupations such as healthcare support, food services, transportation, and manufacturing jobs that could not be performed remotely during lockdowns (28). Consequently, these workers faced increased exposure to the virus. Many residents also rely on public transportation, which further elevates the risk of transmission due to crowding and limited ventilation. Additionally, the high proportion of multigenerational households and densely populated housing in the area made self-isolation and physical distancing more difficult, contributing to faster intra-household spread. Language barriers, digital divide issues, and historic mistrust in health systems may have also impacted health communication and vaccine uptake (65).

These factors collectively elevated the community’s risk of COVID-19 infection, severe outcomes, and delays in receiving preventive and routine healthcare services. To address these issues, public health planners can:

- Implement localized health outreach programs that are culturally sensitive and community-led.
- Expand mobile vaccination and health screening clinics in underserved neighborhoods.
- Increase investment in primary care and preventive services specifically targeting vulnerable areas.
- Develop communication strategies that build trust, address misinformation, and are tailored to the linguistic and cultural needs of the community.
- Integrate social determinants of health frameworks into pandemic preparedness planning to prioritize marginalized populations from the outset.

These measures would not only aid in recovery from the COVID-19 pandemic but also build resilience against future public health crises.

Our study has some limitations. Our data was gathered from only three clinical centers, York Region Public Health Unit (Master No. 2270), Peel Public Health Unit (Master No. 2253), and Toronto Public Health Unit (Master No. 3895), and from limited resources. Our data were gathered and analyzed in aggregate; thus, it may not reflect individual-level social vulnerabilities. Finally, we are not able to understand and analyze the negative impacts of the COVID-19 that will appear only in the long-run. Therefore, it is not possible to measure the impact of such consequences on socially vulnerable communities, at the moment.

Future research should further investigate the longitudinal studies to assess the long-term health impacts of the COVID-19 pandemic on underserved communities such as Jane and Finch. Individual-level data collection, including socioeconomic, demographic, and health behavior factors, would provide a more granular understanding of disparities. Additionally, qualitative studies involving community members could help uncover underlying barriers to healthcare access and vaccination uptake. Evaluating the effectiveness of targeted interventions aimed at supporting socially vulnerable populations during health crises would also be essential to inform proactive, equity-driven responses in future pandemics. Finally, our results indicate that certain FSAs outside the Jane and Finch community, particularly in Etobicoke, Brampton, and parts of Scarborough, also experienced disproportionately high rates of COVID-19 infection, hospitalization, and mortality (Figure 5). These areas warrant further investigation in future research to better understand the underlying factors contributing to these outcomes.

## 5 Conclusion

The LISA significance and cluster analysis indicate that the Jane and Finch community is a hotspot that experienced significantly higher rates of COVID-19 infection, hospitalization, and mortality. This is also confirmed by the statistical *p*-values (*p* < 0.0001) derived from Mann–Whitney U, Wallis-Kruskal, and *t*-tests. Moreover, the Jane and Finch community has had a lower vaccination percentage for first, second, and third doses; nevertheless, it is significant only for the third dosage (*p* = 0.0004). For the first and second dosages of vaccination, in which the p-values are not significant, the Gini index shows perfect equality for all the FSAs. In other words, all the locations have equally administered the first and second dose vaccinations, and there is not much difference between them.

The number of patients across different health parameters, including PPC, cancer screenings, completed IPS, and surgery waitlists decreased more during the COVID-19 pandemic compared to the pre-pandemic period, in the Jane and Finch community. However, this reduction is significant for pap-smear tests (0. 041), and surgery waitlists (0.037). It is worth mentioning that for other health statistics that their p-value is not significant, the Gini index shows perfect equality for all the FSAs, signifying equal burden of the disease among all the neighborhoods.

Our findings highlight the importance of granular, neighborhood-level data to identify communities most at risk during public health crises. In future pandemics, public health agencies and planners should prioritize targeted interventions in communities like Jane and Finch by improving workplace protections for essential workers, ensuring equitable access to vaccines and testing, increasing the availability of multilingual and culturally responsive public health messaging, and investing in housing and transit infrastructure to reduce exposure risks. Tailored outreach programs, developed in collaboration with trusted community organizations, can further support healthcare engagement and reduce disparities in outcomes. The results of this work will help health officials identify the health areas in which the Jane and Finch community, one of the most socially vulnerable localities of the GTA, have suffered more, and ameliorate the damages caused.

## Data Availability

The raw data supporting the conclusions of this article will be made available by the authors, without undue reservation.
