# Supplementary material for: Disproportionate impact of the COVID-19 pandemic on socially vulnerable communities: the case of Jane and Finch in Toronto, Ontario
Source: Front Public Health. 2025 Jun 11;13:1448812. doi: 10.3389/fpubh.2025.1448812 (PMC12189308; doi:10.3389/fpubh.2025.1448812)
Supplement: Supplementary file 1 [file Data_Sheet_1.docx]

Supplementary File 1

# Introduction

Ontario Marginalized Index (ON-Marg) is a metric used for identifying disadvantaged localities in Ontario. ON-Marg is commonly deployed in quantiles, where the first and fifth quantile signify the least and most disadvantaged classes. The Jane and Finch community is one of the most vulnerable neighborhoods of the Greater Toronto Area (GTA) which is composed of 7 Forward Sortation Areas (FSA), namely, M3J, M3M, M9L, M3N, M9M, M3L, and M3K, is classified in the fifth quantile of ON-Marg. In this work, FSAs of three regions in GTA, Peel, York, and city of Toronto, which are classified in the first to fourth quantiles of ON-Marg are compared with Jane and Finch community, in terms of burden of COVID-19 pandemic. Supplementary Table 1 displays all the FSAs that are compared with the Jane and Finch community.

**Supplementary Table 1.** FSA that are compared with the Jane and Finch community.

| M1B | M1E | M1G | M1H | M1J | M1K | M1L | M1M | M1P |
| --- | --- | --- | --- | --- | --- | --- | --- | --- |
| M1R | M1S | M1T | M1V | M1W | M2H | M2J | M2M | M2R |
| M3C | M4A | M4H | M4X | M5A | M5T | M6A | M6E | M6L |
| M6N | M6M | L4T | L6G | M9N | M9R | M9V | M9W | L4X |
| L6T | L6V | M6S | M4T | M5G | M9A | L5C | M1N | M4Y |
| M1X | L3X | L3P | M6B | L4W | L6E | M4C | M4E | M5M |
| M5J | M8Z | L5K | L7B | L7C | M4J | M4S | L5B | L6R |
| L5G | L5T |  |  |  |  |  |  |  |

# Results

Statistical tests indicate that the Jane and Finch community have a significantly higher number of COVID-19 cases, hospitalizations, and mortalities, and a significantly lower third dose vaccination percentage compared to other FSAs under study. Supplementary Figures 1 (a-d) show the distribution of COVID-19 cases, hospitalizations, mortalities, and third dose vaccination percentage, and other FSAs, and Supplementary Figures 2 (a-d) show the histogram of that, respectively. In Supplementary Figure 2 (a-d) the mean value for the Jane and Finch community along with the 95-percentile confidence interval is also displayed.


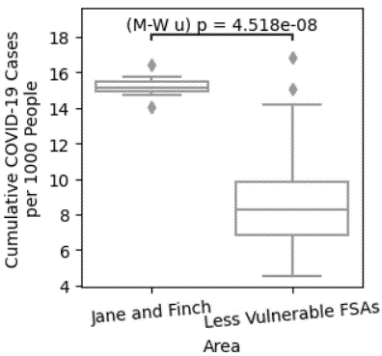

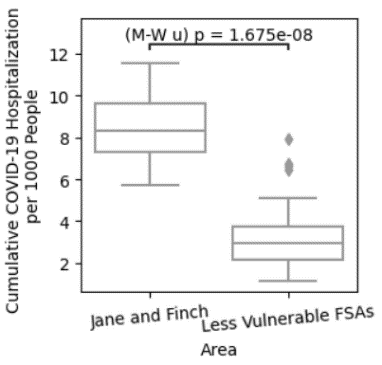

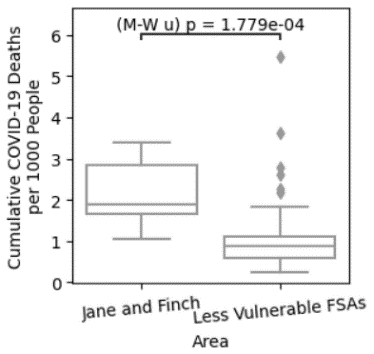

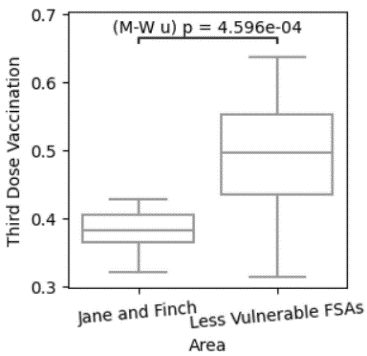


(a) (b) (c) (d)

**Supplementary Figure 1.** Distribution of the number of COVID-19 (a) cases, (b) hospitalizations, and (c) mortalities and (d) percentage of third dose vaccination for Jane and Finch community and other FSAs.


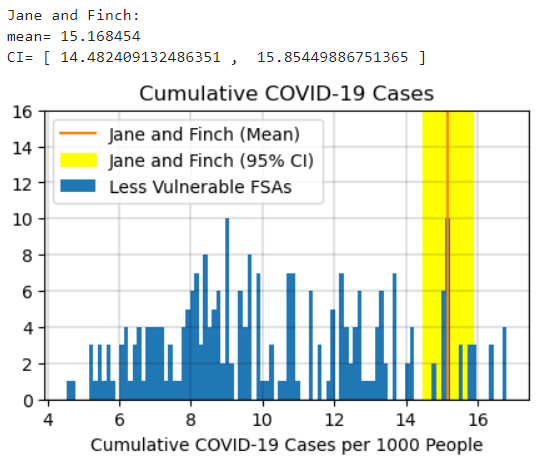

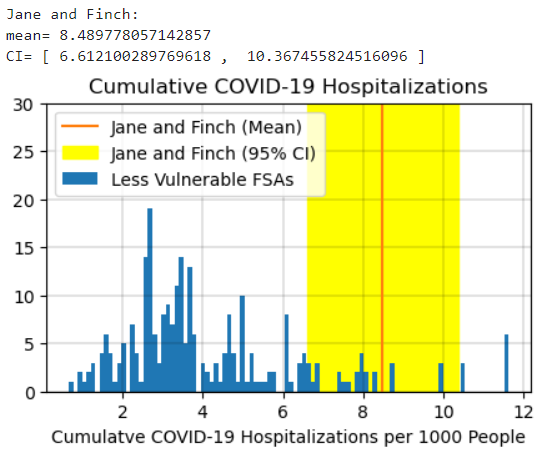


(a) (b)


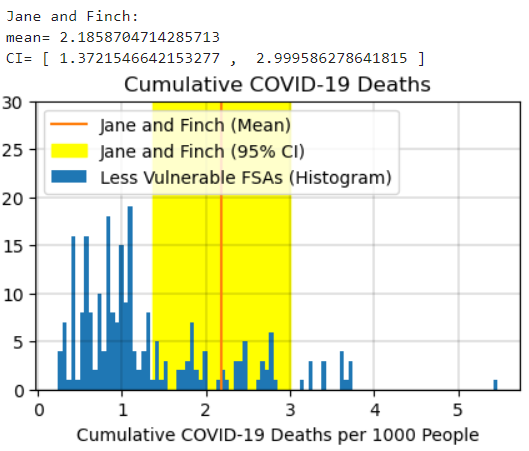

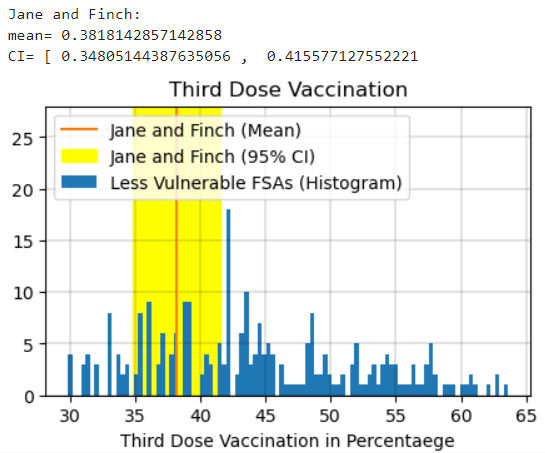


(c) (d)

**Supplementary Figure 2.** The histogram of the number of COVID-19 (a) cases, (b) hospitalizations, and (c) mortalities, and (d) percentage of third dose vaccination.

In addition to COVID-19 statistics, the pap-smear screenings, and surgery waitlists has significantly decreased more during COVID-19 pandemic versus before that for the Jane and Finch community in comparison to other FSAs under study. Supplementary Figures 3 (a, b) show the distribution of pap-smear screening and surgery waitlist reduction during COVID-19 versus before that, and, Supplementary Figures 4 (a, b) show the histogram of that, respectively. Supplementary Figures 4 (a, b) also display the mean and 95-percentile confidence interval of the values for the Jane and Finch community.


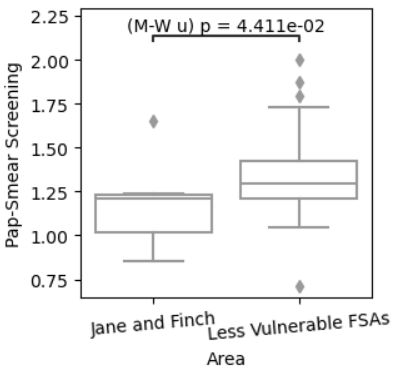

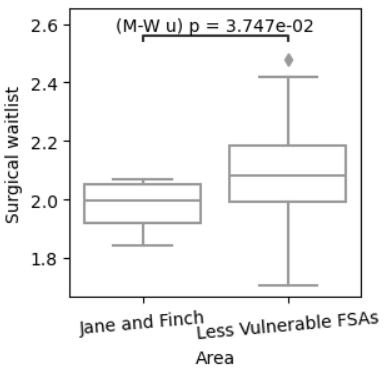


(a) (b)

**Supplementary Figure 3.** The distribution of patient reduction during COVID-19 pandemic versus before that for (a) pap-smear screening, and (b) surgery, for the Jane and Finch community and other FSAs.


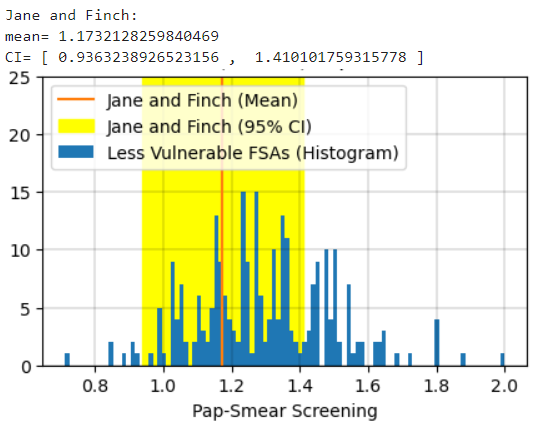

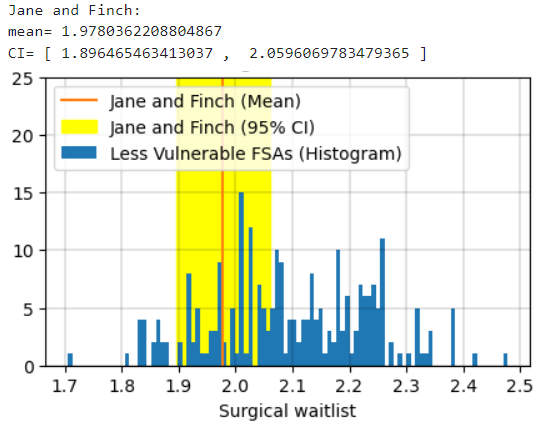


(a) (b)

**Supplementary Figure 4.** The histogram of patient reduction during COIVD-19 pandemic versus before that for (a) pap-smear screenings and (b) surgery.

# Conclusion

Similar to previous crises, disadvantaged and vulnerable communities have suffered a heavier burden of disease during the COVID-19 pandemic. The Jane and Finch community, as one of the most vulnerable neighborhoods in the Greater Toronto Area (GTA) is detected as one of the hotspots that disproportionately have a higher number of COVID-19 cases, hospitalizations, and mortalities. Moreover, for more information, please refer to our manuscript:
